# Modelling Annihilation Properties of Positronium Confined in Nanoporous Materials: A Review

**DOI:** 10.3390/ijms25073692

**Published:** 2024-03-26

**Authors:** Fabrizio Castelli, Giovanni Consolati

**Affiliations:** 1 Department of Physics “Aldo Pontremoli”, Università degli Studi di Milano, Via Celoria 16, 20133 Milano, Italy; 2 Istituto Nazionale di Fisica Nucleare, Sezione di Milano, Via Celoria 16, 20133 Milano, Italy; giovanni.consolati@polimi.it; 3 Department of Aerospace Science and Technology, Politecnico di Milano, Via La Masa 34, 20156 Milano, Italy

**Keywords:** nanoporous materials, positronium lifetime spectroscopy, exchange correlations

## Abstract

Positronium (Ps) is a valuable probe to investigate nanometric or sub-nanometric cavities in non-metallic materials, where Ps can be confined. Accessible experimental measurements concern the lifetime of trapped Ps, which is largely influenced by pick-off processes, depending on the size of the cavity as well as on the density of the electrons belonging to the surface of the host trap. Another relevant physical quantity is the contact density, that is the electron density at the positron position, which is usually found to be well below the vacuum value. Here, we review the principal models that have been formulated to account and explain for these physical properties of confined Ps. Starting with models, treating Ps as a single particle formulated essentially to study pick-off, we go on to describe more refined two-particle models because a two-body model is the simplest approach able to describe any change in the contact density, observed in many materials. Finally, we consider a theory of Ps annihilation in nanometric voids in which the exchange correlations between the electron of Ps and the outer electrons play a fundamental role. This theory is not usually taken into account in the literature, but it has to be considered for a correct theory of pick-off annihilation processes.

## 1. Introduction

Positronium (Ps), the bound state of an electron and a positron [1], is widely used to characterize structural properties of materials. Indeed, its unique behaviour inside matter allows one to obtain information about the material under investigation, using Ps as a probe.

In order to form Ps, positrons need to be implanted inside the medium under study. In metals, positrons annihilate without forming Ps. On the other hand, Ps formation is possible in many different classes of materials, such as zeolites [2], porous structures [3], amorphous and crystalline SiO_2_ [4], polymers [5], glassy materials [6], to quote just a few examples. Ps formation is correlated to material-specific properties, such as the electron density, its internal structure, ionization energy, or positron work function. After a short slowing down in the bulk up to thermal energies [1,7], a positron may catch an electron of the material and install as a Ps atom into regions characterized by low electron density, like free spaces, defects, and vacancies.

If these spaces are not interconnected, Ps will remain confined inside these cavities, even of sub-nanometric size, and there it will annihilate. This process is strongly correlated to the local features of the medium. In a vacuum, the annihilation rate is influenced by the so-called *contact density* $k_{0}=1/8\pi a_{0}^{3}$ (where $a_{0}$ is the Bohr radius), which represents the electron density at the positron [8]. This parameter can be experimentally determined from the magnetic quenching of Ps. On the other hand, in matter the *pick-off* process becomes dominant; with this additional channel the positron inside Ps annihilates with an external electron belonging to the surrounding medium. As a consequence, the lifetime of the long-living Ps component *ortho*-Ps (or in short, *o*-Ps) may be strongly reduced with respect to the one in the vacuum. Nevertheless, it is longer than other lifetimes derived from different annihilation channels, and can be easily separated from them using the PALS (Positron Annihilation Lifetime Spectroscopy) experimental technique [7]. Connecting experimental data with theoretical models featuring the properties of Ps in small voids allows one to get valuable information on (sub)nanometric holes present in the bulk as well as their mean size and electron density near the surface.

The annihilation properties of ground state Ps in a vacuum depend on its internal spin configuration. Indeed, there are two different lifetimes. A short $\tau_{2\gamma }=\lambda_{2\gamma }^{-1}=0.125\;\mathrm{ns}$ for *para*-Ps (or in short, *p*-Ps), the singlet state, and a much longer lifetime: $\tau_{3\gamma }=\lambda_{3\gamma }^{-1}=142\;\mathrm{ns}$ for *o*-Ps, the triplet state. The corresponding annihilation rates are denoted as $\lambda_{2\gamma }$ and $\lambda_{3\gamma }$ [9]. Subscripts 2γ and 3γ remind us that annihilation occurs with emission of two and three quanta, respectively. Confinement of Ps in a hole involves a change in both the short and long components in a PALS spectrum, which mirrors the presence of a possible statistical mixture of Ps states, and signals a possible modification both in spatial wavefunction as well as in the spin configuration.

In this review, we analyse some theoretical modelling of Ps confined in nanoporous matter, which has been proposed to describe the annihilation properties and to find connection with experimental annihilation spectra. After a section presenting common basic expressions for the annihilation rates, we discuss approximate models based on one or two-body description for Ps pick-off processes. Subsequently, we re-examine the problem of Ps confinement in materials showing (sub)nanometric holes by approaching it with a more refined model, in which the exchange interaction of a Ps electron with surrounding electrons is fully considered by means of the methods of symmetry-adapted perturbation theories (SAPT) and local density approximation (LDA).

## 2. General Description of Ps Annihilation in Nanocavities

Considering Ps preferentially localized in low-electron regions like nanocavities, Ps interaction with the electrons belonging to the cavity is modelled in terms of a small perturbation which depends only on the electron density. These surrounding electrons are responsible for the annihilations by pick-off and, in a first approximation, are not correlated to the Ps spatial wavefunction and spin configuration. In fact, the underlying mechanism of pick-off, mixing the fast singlet decay mode with some external electrons during wall collisions, determines the shortening of the *o*-Pslifetime. Therefore, theoretical treatments formulated in this framework consider Ps as a separate *entity* with respect to the environment. Compared to the external electrons, the electron of Ps has a kind of privileged role, discarding the request of full electron indistinguishability, which may be related to the pick-off process. As a matter of fact, complete electron indistinguishability clearly emerges in some materials whose PALS spectrum shows a single lifetime component (the ion ${\mathrm{Ps}}^{-}$ being a straightforward example of a Ps-like system characterized by a single lifetime component). Modelling Ps annihilation taking explicitly into account spin configurations and exchange correlation effects will be the subject of a later section.

Another important phenomenon for Ps physics, experimentally observed in many materials [8,10], is the decrease in the contact density, that is, a lowering of the electron density at the positron position with respect to the one in a vacuum. To describe this effect, a relative contact density parameter $k_{r}$ is introduced; it is defined as the probability to find the electron of Ps at the positron position in units of the value $k_{0}$ in vacuo. This parameter is usually related to a deformation of the Ps wavefunction inside the host cavity [11,12].

The most general expressions describing the Ps triplet/singlet measurable annihilation rates in porous matter, as proposed in the literature [13], are built by considering in the same way intrinsic and pick-off contributions:

(1) $$\begin{array}{cc} \lambda_{t}\; & =\;k_{r}\;\lambda_{3\gamma }+\lambda_{\mathrm{po}}\;,\\ \lambda_{s}\; & =\;k_{r}\;\lambda_{2\gamma }+\lambda_{\mathrm{po}}\;. \end{array}$$

where $\lambda_{t}$ and $\lambda_{s}$ are the annihilation rates for *o*-Psand *p*-Ps, respectively, while $\lambda_{\mathrm{po}}$ is the pick-off annihilation rate, identical in both equations, naturally being a surface process. The intrinsic contribution is characterized by $k_{r}$, the relative contact density parameter. In the simplest implementations, every model assumes that the pick-off rate $\lambda_{\mathrm{po}}$ is related to a geometrical probability $P_{\mathrm{out}}$ to find Ps outside the *internal* free-space region which defines the nanocavity:

(2) $$\lambda_{\mathrm{po}}=P_{\mathrm{out}}\bar{\lambda }\;,$$

where $\bar{\lambda }$ is defined as the spin averaged *o*-Psand *p*-Psannihilation rates in vacuum:

(3) $$\bar{\lambda }\;=\;\frac{1}{4}\lambda_{2\gamma }+\frac{3}{4}\lambda_{3\gamma }\;=\;2.01\;\;\;{\mathrm{ns}}^{-1}\;,$$

since in vacuo the probability formation of *p*-Pswith respect to *o*-Psis 1/3. Therefore, $\bar{\lambda }$ does not depend on the electronic properties of the host cavity; these are accounted for in the expression of $P_{\mathrm{out}}$, which is a function of the geometrical parameters of the adopted model. They are selected in order to obtain the best agreement with the pick-off annihilation rate as given by the experiment.

Some models make the simple assumption of $k_{r}=1$ [14,15,16], equivalent to guess that confinement does not influence the intrinsic Ps annihilation rates. More refined models introduce different prescriptions concerning the correct way to tackle the problem of Ps located in the internal or in surface regions of the hosting cavity. For instance, in [17,18], Ps is supposed to annihilate inside the hole with intrinsic vacuum rates. Conversely, pick-off is the dominating annihilation channel in the surface region. Within this picture, it is assumed that $k_{r}=1-P_{\mathrm{out}}$, which in fact represents the probability that Ps is found in the internal region of the cavity.

## 3. One-Particle Theoretical Models

To give a quantitative description of Ps behaviour in materials with nanoholes, one-body models are the simplest ones. They consider Ps as a structureless single quantum particle. Simple trapping quantum wells (finite or infinite) are introduced to account for the interaction potentials between Ps and the surrounding. The idea of Ps trapping was introduced for the first time by Ferrell [19], to give a reason for the unforeseen long *o*-Pslifetime in liquid He. He proposed that Ps may form a nanoscopic bubble around itself, generated by the exchange repulsion between the Ps–electron and the external electrons of the helium atoms. The equilibrium radius of the bubble was estimated by the order of the Ps Bohr radius $a_{Ps}$, which is equal to $2a_{0}$.

### 3.1. The Tao–Eldrup (TE) Model

**Figure 1:**
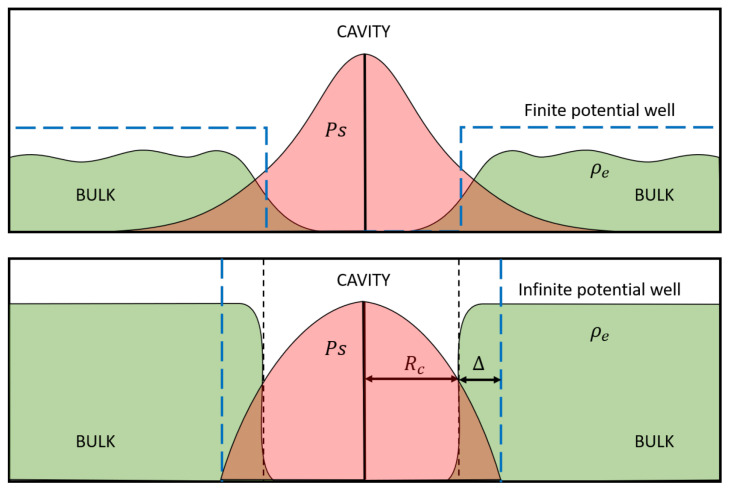
Graphic explanation of TE approximation. A Ps trapped into a finite potential well (**top** frame) is transformed into a particle confined into an infinite potential well (**bottom** frame).

In 1972, Tao proposed [20] a model relating the typical sizes of the cavity inside matter hosting Ps with the lifetime of this last. Modified a few years later by Eldrup et al. [21], it became the most popular one-particle model. Tao transformed Ps localization inside a spherical volume of radius $R_{c}$ into a confinement within a potential well of infinite depth of size $R_{c}+\Delta$, as depicted schematically in Figure 1.

Due to the infinite strength of the confining potential, the wavefunction of Ps has to become zero at a chosen boundary. Concerning the external electron density profile $\rho_{e}$, it is described by a step function which is zero inside the hole and assumes a constant maximum value $\rho_{e}∼k_{0}$ (generally equal to the vacuum contact density) outside. If the Ps wavefunction boundary coincides with the radius $R_{c}$, there is no overlap between Ps and the electrons of the cavity. The consequence would be that Ps decay by pick-off could not occur. To avoid this nonsense, Tao supposed the Ps boundary to be located at a layer of thickness Δ surrounding the hole, which is, therefore, the interacting region.

With this position the ground state Ps wavefunction has the following expression:

(4) $$\Psi_{\mathrm{TE}}\left(R\right)\;=\;\frac{1}{\sqrt{2\pi (R_{c}+\Delta )}}\frac{\sin\left(\pi R/(R_{c}+\Delta )\right)}{R}\;.$$

It follows that the probability $P_{\mathrm{out}}$ is given by:

(5) $$P_{\mathrm{out}}\;\equiv \;4\pi \int_{R_{c}}^{R_{c}+\Delta }{\left|\Psi_{\mathrm{TE}}\left(R\right)\right|}^{2}R^{2}\;dR\;,$$

and the pick-off decay rate results:

(6) $$\lambda_{\mathrm{po}}\;=\;\bar{\lambda }\;\left(1-\frac{R_{c}}{R_{c}+\Delta }+\frac{1}{2\pi }\sin\frac{2\pi R_{c}}{R_{c}+\Delta }\right)\;.$$

This last formula gives a relationship between two geometrical quantities, $R_{c}$ and Δ, generally unknown, and the annihilation rate, which can be supplied by the experiment. The thickness Δ is assumed to be a universal constant whose value can be evaluated by fitting the TE model on materials whose cavity sizes are known from different experiments. The value Δ=0.166 nm was at first obtained from materials whose cavity radii were in the range $R_{c}$ = 0.32–0.38 nm and corresponding *o*-Pslifetimes from 2.45 to 3.2 ns. The validity of the TE model is limited to nanometric cavities ($R_{c}<2.5$ nm) [22] where thermal energy is not sufficient to populate excited states of Ps confined in the well.

In the TE model, the annihilation rate is completely independent of the chemical properties of the material and becomes a question of geometry. The interest of the chemist community, combined with the improvement of the sensitivity of the PALS technique led to the question of whether Ps trapped in a cavity could not only give information about the geometry of the hole but also shed light on the chemical features of the medium surrounding Ps. In this connection, various extensions of the TE model have been put forward along the years, sometimes including additional parameters which have to be determined somehow.

### 3.2. Some Extensions of the TE Model

Dutta [23] introduced a potential well of finite height $U_{0}$. There are various advantages in this approach. First of all, the removal of the infinite potential barrier, which is unphysical and would have the consequence that a Ps would disappear for a cavity radius sufficiently small. Secondly and more important, there is no longer need for the geometrical layer Δ. Indeed, now the Ps wavefunction can overcome the hole wall and spread into the bulk, where the pick-off process occurs. Moreover, for both the potential and the electron density, Dutta considers a smooth function instead of the step potential assumed in the TE model, but at the price of introducing other parameters in the model.

The extension of the TE model for increasing void dimension, where more energy levels appear in the potential well and the pick-off rate is thermally averaged over populated states, was proposed in [14]. On a similar basis, also the so-called rectangular TE [4] extended the original TE model by replacing the spherical geometry with rectangular voids, with the advantage of avoiding cumbersome calculations by keeping physical simplicity. These two formulations can be easily adapted to various geometries of the voids and also allow us to deal with temperature effects. Once calibrated, these models were able to successfully fit PALS results for a broad range of void sizes, up to several hundredths of nm, as well as to explain the temperature dependence of PALS experiments.

### 3.3. A Classical Modellization

Finally, we mention a model proposed by Wada and Hyodo [24] for the estimation of the size of voids which is independent of their shape. These authors consider a cavity of generic geometry in which Ps (with finite size) is bouncing inside. This classical scheme is valid as long as the mean free path of Ps ${\bar{L}}_{Ps}$ between collisions with the walls of the hole are much larger than $\lambda_{Ps}=h/\sqrt{4\pi K_{B}T}$, which is the thermal Ps de Broglie wavelength, amounting to 3.05 nm at 300 K. For a finite-size Ps, ${\bar{L}}_{Ps}$ depends on two parameters: $\bar{\Delta }$, which define its effective average size, and $\bar{L}=4V/A$, the mean free length of the void, *V* and *A* being the volume and the surface of the cavity, respectively (in particular, $\bar{L}=4V/R_{c}$ for a spherical cavity), by means of the obvious relation ${\bar{L}}_{Ps}\;=\;\bar{L}-\bar{\Delta }$. The *o*-Pspick-off rate is then simply obtained as the product of the pick-off annihilation probability per collision with the wall of the hole $P_{A}$, and the collision frequency $v_{\mathrm{th}}$/${\bar{L}}_{Ps}$, with $v_{\mathrm{th}}$ being the thermal velocity:

(7) $$\lambda_{\mathrm{po}}\;=\;P_{A}\;\frac{v_{\mathrm{th}}}{\bar{L}-\bar{\Delta }}\;.$$

For a suitable choice of the free parameter $\bar{\Delta }$, Equation (7) gives the same results of the TE model for an arbitrary small value of the cavity radius. The Wada and Hyodo model provided good agreement with the rectangular version of the former one for a cavity of a size bigger than $\bar{L}>1.28$ nm, therefore demonstrating that the pick-off process in mesoporous materials is primarily correlated to the V/A ratio.

## 4. Two-Particle Theoretical Models

The increasing interest in the PALS experimental technique and the large production of relative data suggest the single-particle approximation with some new modelization is overcome. The logical path of extending theoretical TE-like models is of course to introduce the positron and the electron in Ps separate degrees of freedom [11,25,26]. In this way, Ps is described by a two-particle wavefunction Ψ(p,e), where *p* and *e* represent the spin–spatial coordinates of the positron and electron, respectively, at variance with the TE wavefunction that depends on a single spatial variable. Other treatments of the two-particle bound system were considered, for example, with ab initio techniques [27], but in view of the huge computational effort required they are not usually employed.

Generally speaking, the two-body approach for confined Ps can be formulated in a manner of describing both the centre of the mass wavefunction and its internal structure. Moreover, a two-body model is the simpler and appropriate way for determining the contact density parameter *k*, which governs the annihilation processes [8,10]. Describing theoretically any variation of the contact density parameter *k* with respect to its vacuum value $k_{0}$ can be simply obtained by this general expression:

(8) $$k\;=\;\int \;|\Psi \left(r_{p},r_{e}\right){|}^{2}\delta \left(r_{p}-r_{e}\right)\;d^{3}r_{p}\;d^{3}r_{e}\;,$$

where the integration spans the entire configuration space, and it is performed on the part of the wavefunction which depends only on the spatial coordinates $r_{p}$ and $r_{e}$ for the positron and electron, respectively. The spin part of the wavefunction is usually not considered because Ps community is generally interested and focused on *o*-Ps.

As usual, in any two-particle system, Ps wavefunction can be reformulated in terms of functions of the relative coordinate $r=r_{p}-r_{e}$ and of the centre of mass coordinate $R=(r_{p}+r_{e})/2$. In the cases in which the problem under study is separable, the wavefunction can be factored as

(9) Ψ(R,r)=Φ(R)φ(r),

and the k parameter is calculated straightforwardly by the following formula:

(10) $$k\;={\;\int \;|\Psi \left(R,0\right)|}^{2}\;d^{3}R\;.$$

The original multiparticle problem is reduced to a simpler one in the two-body models, similarly as for one-body models, by introducing an effective potential for the interaction with the material. This potential is usually obtained from a macroscopic approach, hence naturally introducing some new free parameters that have to be experimentally established. To determine the Ps wavefunction and to find the associated ground state, numerical methods are usually employed on the resolution of the total hamiltonian *H* of the system, which is a function of these appropriate potentials. For this purpose, two main techniques are considered, namely the quantum Monte Carlo (QMC) and the variational method (VM). We now review some simple two-particle models which can be solved by using the variational approach.

### 4.1. The Compressed Ps Model

A simple model that retains some characteristics of the one and two-particle models is presented in [28]. In their approach, the focus of the authors is on the relative coordinate r instead of the more traditional centre of mass one. The vanishing boundary condition that in TE-like models imposes the confinement acting solely on the centre of the mass wavefunction in this proposal is instead imposed on the relative wavefunction φ(r). By selecting a spherical symmetric cavity for simplicity, the centre of mass is considered stuck in the centre of the hole, so that one can write the factorization $\varphi \left(r\right)=R\left(r\right)Y_{lm}\left(\theta ,\phi \right)$ as usual. Moreover, the Hydrogen-like Schrödinger equation is obtained for the variable u(r)=rR(r):

(11) $$\frac{d^{2}u}{d\rho^{2}}\;+\left[-\frac{1}{4}+\frac{n}{\rho }-\frac{l(l+1)}{\rho^{2}}\right]\;=\;0\;,$$

n,l,m being the well-known quantum numbers, ρ=2κr with $\kappa =\sqrt{\mu |E|}/\hbar$ and $n=1/a_{Ps}\kappa$ (μ being the reduced Ps mass, while $a_{Ps}$ is the Ps Bohr radius $=2a_{0}$). The solution of this equation is then looked in the familiar form $u\left(\rho \right)=\rho^{l+1}e^{-\rho /2}F\left(l,\rho \right)$, but the fundamental difference as compared to the free-Ps case is that the series expansion for *F* has to vanish at $r=2R_{c}$ instead of vanishing to infinity, due to the boundary condition. This is because the maximum radial distance *r* has to be equal to the diameter of the trapping site $2R_{c}$ with the assumption of the centre of mass located at the cavity centre. As a consequence, the principal quantum number *n* is no longer an integer, but assumes real values. After a numerical evaluation of *n*, also the corresponding shift of the energy levels *E* can be obtained.

A formula for the relative contact density is easily obtained as

(12) $$k_{r}\;=\;\frac{1}{n^{2}A^{2}}\;,$$

where the quantity *A* is the normalization factor of the wavefunction, depending on the cavity radius $R_{c}$ as well as *n*. Within this procedure, and taking l=0 for the ground state, a relationship is obtained connecting $R_{c}$ with both *n* and $k_{r}$. As a matter of fact, only for $2R_{c}<0.3$ nm the quantum number *n* differs appreciably from unity, and it is observed that the resulting relative contact density behaves opposite to the cavity radius: it tends to increase when $R_{c}$ decreases. Therefore, the findings of this simple model contrast to the observed swollen Ps, because it demonstrates that the confinement alone inevitably leads to a compressed state. It is clear that some other effects acting on the contact density have to be considered in the construction of more realistic yet complicated models.

### 4.2. The Springs Model

A two-particle model which is exactly solvable was proposed by McMullen and Stott [29]. In that so-called spring model, the interactions acting on the positron and on the electron of the Ps atom are described as harmonic potentials, appropriately chosen for each particle. Furthermore, such potentials have the role of confining the Ps within a spherical hole in the first realization of the model. The relative hamiltonian is the following:

(13) $$H\;=\;-\frac{\hbar^{2}\;\nabla_{r_{p}}^{2}}{2m_{p}}\;-\frac{\hbar^{2}\;\nabla_{r_{e}}^{2}}{2m_{e}}\;+\frac{1}{2}\kappa {\left(r_{p}-r_{e}\right)}^{2}+\frac{1}{2}k_{+}r_{p}^{2}+\frac{1}{2}k_{-}r_{e}^{2}\;,$$

where for definiteness a harmonic attraction with elastic constant κ replaces the Coulomb interaction between the electron and the positron. The other harmonic terms on the left side describe external fields acting separately on the two particles. They can be either attractive or repulsive depending on physical requirements. Positron attraction towards the ions of the surrounding cavity would be described by a negative $k_{+}$, while a realistic choice of $k_{-}$ is expected to be positive, because of the Pauli exchange repulsion of the Ps–electron with the outer close-shell electrons. In the centre of mass reference frame, the solution of the Schrödinger equation associated with the bare hamiltonian (that is without the terms with $k_{+}$ and $k_{-}$) is straightforward

(14) $$\Psi_{0}\left(r,R\right)\;=\;\frac{1}{\sqrt{\Omega }}\;e^{iK\cdot R}\;\phi_{n}\left(r\right)\;,$$

where $\phi_{n}\left(r\right)$ is a spherically symmetric simple harmonic oscillator state, represented by quantum numbers $n=(n_{x},n_{y},n_{z})$.

An important question concerns the boundary condition at the vacuum limit of free-Ps. For example, with the aim of recovering the free-Ps dipole polarizability, the authors find that the value of κ must fulfil the condition:

(15) $$\kappa \;=\;\frac{e^{2}}{36a_{0}^{3}}\;.$$

However, other choices are possible. Indeed, if one is interested to recover the vacuum contact density $k_{0}$, the condition for κ is given by the formula $\kappa =\pi^{2/3}\hbar^{4}/\left(8ma_{0}^{4}\right)$.

Under the choice of Equation (15) and with a suitable set of transformations, the eigenstates of *H* were found, and in particular the ground state reads

(16) $$\Psi_{H}\left(r,R\right)\;=\;{\left(\frac{{\left(k_{u}k_{v}\right)}^{1/4}}{\pi }\right)}^{3/2}\;e^{-(\sqrt{k_{u}}u^{2}+\sqrt{k_{v}}v^{2})/2}\;,$$

where the functions *u* and *v* are given explicitly by

(17) $$\begin{array}{cc} u\; & =\;\sqrt{2m}\;R\;\sin\theta \;+\sqrt{\frac{m}{2}}\;r\;\cos\theta \;\\ v\; & =\;\sqrt{2m}\;R\;\sin\theta \;-\sqrt{\frac{m}{2}}\;r\;\cos\theta \; \end{array}$$

while $k_{v}$ and $k_{u}$ are given by:

(18) $$k_{u/v}\;=\;2\kappa +k_{-}+k_{+}\pm \sqrt{4\kappa^{2}+{\left(k_{+}-k_{-}\right)}^{2}}\;.$$

As expected, it is found that if $k_{-}$ increases, the Ps–electron is more confined, leading to a compressed Ps and to a higher contact density. On the other side, if $k_{+}$ becomes more negative, the positron is more polarized towards the medium, leading to an effect of swelling of the Ps, and to a lower contact density. Between these limits, it is possible to find a condition in which the two effects precisely cancel, that corresponds to the particular line in the parameters space where $k_{r}=1$. A simple interpretation about the physical origin of $k_{\pm }$ and of the significance of the results, is suggested by the authors in the common case of Ps confined in a spherical cavity inside a dielectric. Image forces, attracting both the positron and the electron towards the cavity walls, can not overcome the strong repulsive exchange forces in the case of the Ps–electron. On the contrary, the positron is induced to diffuse in the outer electronic shell. This description is in agreement with the findings of another model of confined Ps due to Stepanov, which is the subject of the next subsection.

### 4.3. The Bubble Model

To go beyond TE like models for the study of Ps annihilation, a successful attempt is proposed by Stepanov et al. [25,30], especially facing the case of Ps in liquids. In their approach, Ps is described as a two-body atom confined inside a bubble created by itself. The formation of this bubble is essentially related to two phenomena. Primarily, the influence of the exchange repulsion between electrons, as in the old proposal of Ferrel [19]. Secondarily, and with the same importance, the action of the Coulombic attractive force between the positron–electron pair, which in fact is enhanced in the inner of the bubble due to the absence of the dielectric screening of the surrounding material. The hamiltonian of this *Bubble Model* is constructed by introducing two independent potential terms for the positron and the electron, respectively, and adding a suitable Coulomb potential $U_{c}$ for their mutual interaction within the polarizable medium:

(19) $$H\;=\;-\frac{\hbar^{2}\;\nabla_{r_{p}}^{2}}{2m_{p}}\;-\frac{\hbar^{2}\;\nabla_{r_{e}}^{2}}{2m_{e}}\;+U_{+}\left(r_{p}\right)+U_{-}\left(r_{e}\right)+U_{c}\left(r_{p},r_{e},R_{c},\epsilon \right)\;,$$

where ϵ is the relative dielectric permittivity of the medium, while $R_{c}$ is the bubble radius.

The independent potentials $U_{+}$ and $U_{-}$, which constitute the main novelty of this bubble model, are simply modelled by using the work functions for the positron $\phi_{+}$ and for the electron $\phi_{-}$:

(20) $$\begin{array}{ccc} U_{+}\left(r_{p}\right) & =\left\{\begin{array}{cc} 0\; & \;r_{p}<R_{c}\\ -\phi_{+}\; & \;r_{p}>R_{c} \end{array}\right.\;\;U_{-}\left(r_{e}\right) & =\left\{\begin{array}{cc} 0\; & \;r_{e}<R_{c}\\ -\phi_{-}\; & \;r_{e}>R_{c} \end{array}\right. \end{array}$$

This formulation of these potentials has the following physical meaning: if the work function is positive, which is if it requires a positive work to extract a particle from the material, then the particle in question will be attracted towards the medium and vice versa. The Coulomb potential $U_{c}$ takes into account the polarization of the medium and, in the not straightforward geometry of a uniform dielectric medium containing a spherical void (the bubble), can be determined for every inter-particle distance $r=|r_{p}-r_{e}|$ by solving the Poisson equation, resulting in a complicated function of Legendre polynomials.

To solve the Schrödinger equation with the above hamiltonian for obtaining the ground state solution, a variational method is employed by starting with the trial wavefunction:

(21) $$\Psi_{Step}\left(r,R\right)\;=\;\frac{\exp(-r/2a-R/2b)}{8\pi \sqrt{a^{3}b^{3}}}\;,$$

with *a* and *b* variational parameters. Minimizing the energy, the best solutions for a,b are found so that it is possible to evaluate the contact density using Equation (10). The pick-off annihilation rate is calculated with the simple formula of Equation (2) with precisely the probability $P_{out}^{bubble}$ of finding the positron outside the bubble wall as

(22) $$\lambda_{\mathrm{po}}\;=\;\bar{\lambda }\;P_{out}^{bubble}\;=\;\bar{\lambda }\;\int_{r_{p}>R_{c}}\;{\left|\Psi \left(r_{p}-r_{e}\right)\right|}^{2}\;d^{3}r_{p}\;d^{3}r_{e}\;.$$

All the results that are extracted from the bubble model depend on the following four parameters: $R_{c},\phi_{+},\phi_{-}$ and ϵ, which can be determined with independent experimental measurements. In general, the parameters ϵ and $\phi_{-}$ are easily accessible, but this is hardly true for the others, $R_{c}$ and $\phi_{+}$. In the particular case of liquids, it is customary to use a further equilibrium condition allowing one to relate $R_{c}$ to the macroscopic surface tension σ [25].

It is important to note that in the small radius limit it is straightforward to derive that the relative contact density obeys the relation $k_{r}=k/k_{0}\to 1/\epsilon^{3}$. This relation indicates that, among other models, the bubble model is capable of directly explaining the lowering of the contact density. In the opposite limit of large cavities, the value of the contact density in the vacuum is regained, as expected. Regarding the pick-off annihilation and its relation with the bubble radius, the predicted results vary independently of the choice of $\phi_{+}$; with a suitable choice of parameters the simple TE results can be reproduced.

A possible drawback of this theory is that, for an accurate description of polarization effects in the medium, the variational wavefunction of Equation (21) is retained to be too simple. On the other hand, in the description of confined Ps the idea of considering the dielectric properties of the medium is not new. This kind of approach was already studied in the past with powerful numerical methods, for example, by Bug et al. [26] with Quantum Monte Carlo simulations. Surprisingly, the differences between their results and those of the bubble model are very little, hence the physical simplicity of the present description can be fully appreciated.

### 4.4. A Formal Two-Particle Model for Ps Confinement in Nanoporous Materials

So far, we have described some of the main two-particle models, which can be applied to the study of Ps in porous materials. But from the point of view of the experimental community, these models are not fully satisfactory. This is because they are often not able to foretell the observed lowering of the contact density, or they are too complicated having to rely on extensive material-specific calculations, therefore losing simplicity and immediate physical insight.

To try to overcome these drawbacks, for the description of Ps confined in nanopores in [11,12] a formal two-particle model inspired by the generality of the TE–like approach is proposed, and by the consideration of specific chemical properties for the surrounding material. This model starts from a fundamental observation over the confining potential acting on Ps. As a matter of fact, the confinement is a net result of two different and independent contributions, which act separately on the Ps constituent particles, the positron, and the electron. Regarding the positron, theoretical models [31,32,33] and experimental observations, for example, in silica [34], find a positive value for the positron work function. This suggests that the positron may be attracted toward the material; therefore, it is not subject to a strict confinement. On the other side, the Ps–electron is affected by a repulsive Coulomb interaction at short distances with bulk electrons, and moreover to strong Pauli exchange forces [11,19,35]. Therefore, the Ps–electron tends to prevent a large overlap between the Ps itself and bulk electrons, inducing the known confinement behaviour. This picture can be practically supported by the observations in scattering experiments of Ps over noble gas atoms or molecules. In fact, in spite of the differences in mass and charge, the total Ps cross-section is found to be practically identical to that of a bare electron. This clearly indicates that electron exchange forces dominate short-range interactions [36,37].

This interpretation of the Ps-confining behaviour can be formalized as follows, obtaining a model that is able to describe correctly the lowering of the contact density. By comparing experimental data on this quantity and on the pick-off annihilation rate with the findings of this model, promising results can be obtained enlightening the underlying connections between these quantities with the size of the cavity and the positron work function, which is of immense importance for the experimental community.

For definiteness, we consider a spherical cavity of radius $R_{c}$ centred on the axes origin and, as usual, define relative $r=r_{p}-r_{e}$ and centre of mass coordinates $R=(r_{p}+r_{e})/2$. The Hamiltonian of the confined positron–electron system assumes the following form:

(23) $$H\;=\;-\frac{\hbar^{2}}{4m}\nabla_{R}^{2}-\frac{\hbar^{2}}{m}\nabla_{r}^{2}-\frac{e^{2}}{\left|r\right|}+V_{conf}+V_{bulk}\;,$$

where *m* is the electron/positron mass. The interaction with the surrounding medium is described by the two potentials $V_{conf}$ and $V_{bulk}$. In particular, the confining potential acting only on the Ps–electron can be taken as an infinite well:

(24) $$\begin{array}{cc} V_{conf}\left(r,R\right) & =\left\{\begin{array}{cc} 0\; & \mathrm{if}\;|r_{e}|<R_{c}\\ \infty \; & \mathrm{if}\;|r_{e}|>R_{c} \end{array}\right. \end{array}$$

where $r_{e}=R-r/2$ is the electron position. The condition expressed in Equation (24) defines a proper geometrical domain in the R,r space, out of which the two-particle wavefunction is constrained to be simply zero.

The interaction potential due to the bulk is acting only on the positron and, in the first approximation, it is equal to the opposite of the work function, with the value taken deep inside the bulk for definiteness, while in the inner of the confining cavity it is considered zero. Moreover, following the TE modelling prescription, the presence of a surface layer of thickness Δ surrounding the cavity has to be taken into account. This layer represents the spatial region between the outermost accessible position to the Ps–electron and the zone of the external medium where the properties of a free positron are predominant. The explicit form of the potential reads

(25) $$\begin{array}{cc} V_{bulk}\left(r,R\right) & =\left\{\begin{array}{cc} 0\; & \mathrm{if}\;|r_{p}|<R_{c}+\Delta \\ -\phi_{+}\; & \mathrm{if}\;|r_{p}|>R_{c}+\Delta \end{array}\right. \end{array}$$

where $\phi_{+}$ is the (positive) work function. The form of this potential is in fact a generalization of what is usually derived with DFT theories and calculations in metals [33]. Reasonable values of Δ are of the order of the bond length between atoms, hence in the range 0.1–0.2 nm. Because it can be seen that with different choices of Δ the results of the model undergo negligible differences, it is possible to fix this parameter at the commonly accepted value of Δ=0.166 nm following [20].

For a solution of the time-independent Schrödinger equation with the hamiltonian of Equation (23), it must observed that the centre of mass is not forced to be confined inside the cavity, but can be also be located outside the cavity with $R>R_{c}$, even if the electron is strictly confined with $|r_{e}|<R_{c}$. As usual, the two-particle wavefunction can be represented in a factored form. Avoiding the introduction of additional geometrical parameters, a convenient choice is a plane wave-like factor for the centre of mass motion, while the relative-motion part $\tilde{\varphi }$ takes into account the following confinement:

(26) $$\Psi \left(r,R\right)\;≃\;\frac{1}{\sqrt{\tilde{V}}}e^{iK\cdot R}\;\;\;\tilde{\varphi }\left(r,R\right)\;,$$

where $\tilde{V}$ is a suitable normalization volume. Here, the function $\tilde{\varphi }\left(r,R\right)$ depends parametrically on *R* by spherical symmetry while retaining the coupling between the coordinates (relative and centre of mass) in the geometrical domain defining the confinement effects. Suitable formulas for $\tilde{\varphi }$ can be easily determined in some limiting situations; the main ones are in the centre of the hole and deep inside the bulk. Then, general expressions for $\tilde{\varphi }$ can be approximatively constructed on different *R* intervals as a piecewise continuous function.

Indeed, if *R* lies deep inside the cavity, it is expected that the ground state $\tilde{\varphi }$ resembles the normalized hydrogen-like vacuum solution:

(27) $$\tilde{\varphi }\left(r,R\to 0\right)≃\varphi_{0}\left(r,\theta ,\phi \right)=\left(2/\sqrt{4\pi }\right)\;a_{Ps}^{-3/2}\;e^{-r/a_{Ps}}$$

where $a_{Ps}$ is the Ps Bohr radius. As *R* approaches the cavity wall, this wavefunction must slowly change towards a different outermost function $\tilde{\varphi }\left(r,R∼R_{c}\right)≃\varphi_{wall}\left(r\right)$, which is strongly deformed and polarized with respect to simpler functions satisfying spherical symmetry [12]. This is due to the electron position constraint, which resets the two-particle wave function outside the geometrical domain determined by Equation (24).

**Figure 2:**
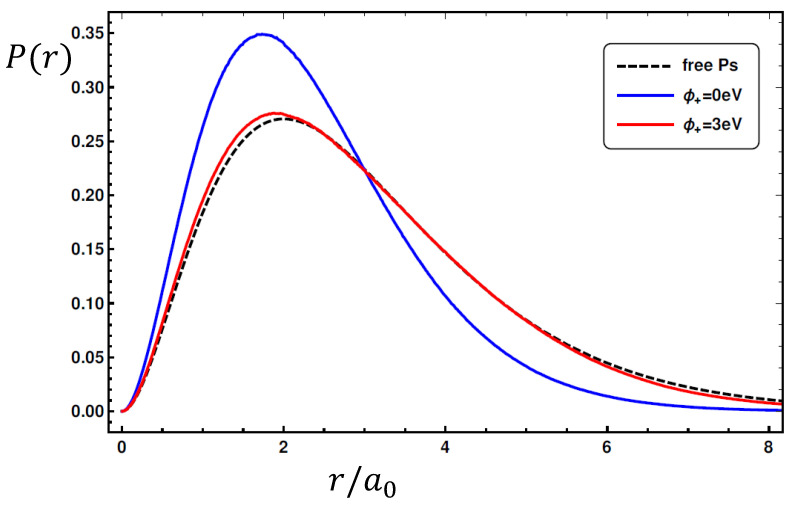
The radial distribution function P(r) of the positron–electron relative distance *r* (normalized on $a_{0}$), for a cavity of $R_{c}=0.17$ nm. The black dashed line is the 1S solution for the free-Ps, the blue line corresponds to the vanishing bulk potential $\phi_{+}=0$ eV, and the red line shows the effect of a nonzero bulk potential $\phi_{+}=3$ eV.

To illustrate an example of the effects of the two potentials over the ground state relative wavefunction $\tilde{\varphi }$, in Figure 2 we plot the radial probability distribution $P\left(r\right)=|r\;\tilde{\varphi }{\left(r,R\right)|}^{2}$ where *r* is the positron–electron relative distance, in the specific case in which the electron is located near the wall (with $|r_{e}|≅R_{c}$), and where the wavefunction is essentially the outermost part $\varphi_{wall}\left(r\right)$. The calculation is performed for a cavity of radius $R_{c}=0.17$ nm to make the confining effect well evident, and for two different positron work functions. In the figure, the black dashed line represents the free-Ps 1S relative distribution, while the blue line represents the effect of the confining potential (at zero positron work function). In this case, one has a squeezing in the radial distribution, with a consequent enhancing of the contact density value with respect to the free case, similarly to the findings of the compressed Ps model [28]. The red line shows the effect of the positron work function in the bulk potential, which is opposite to the above behaviour: the stronger attractive potential acting on the positron due to the higher value of the positron work function tends to swell the wavefunction, with a consequent lowering of the contact density.

As shown in [11], from the need of a unique value of the wavefunction in r=0, one has $\varphi_{wall}\left(0\right)\;=\;\lim_{r\to 0}B\;\varphi_{0}\left(r\right)$, with a suitable normalization coefficient *B*, which can be determined as a function of $\phi_{+}$ and Δ. As expected, it turns out that B<1 due to a lower value of the relative wavefunction with respect to that in vacuum near the wall, because of stretching of the external part towards the medium. Note that in the limit of free-Ps one has of course B=1, and the normalization volume $\tilde{V}$ turns out to be approximatively equal to $4\pi R_{c}^{3}/3$ [11].

Now, without lacking of generality and under the hypothesis that the bulk polarization effects on the Ps wavefunction are important only within a shell of thickness $a_{Ps}$ below the cavity wall (it is unlikely that polarization effects operate over a larger range), a simple piecewise representation for $\tilde{\varphi }\left(r,R\right)$ can be built in the following form:

(28) $$\tilde{\varphi }\left(r,R\right)≃\left\{\begin{array}{cc} \varphi_{0}\left(r,\theta ,\phi \right)\; & \mathrm{if}\;0\leq R<R_{c}-a_{Ps}\\ \varphi_{wall}\left(r,\theta ,\phi \right)\; & \mathrm{if}\;R\geq R_{c}-a_{Ps} \end{array}\right.$$

Having written the two-particle wavefunction in the separable form of Equation (26), the contact density can be calculated directly as in Equation (10):

(29) $$k\;=\;\frac{1}{\tilde{V}}\;\int_{0}^{R_{c}}\;{\left|\tilde{\varphi }\left(0,R\right)\right|}^{2}\;d^{3}R\;\;,$$

where, by using Equation (28) one has

(30) $$\begin{array}{cc} |\tilde{\varphi }{\left(0,R\right)|}^{2} & =\left\{\begin{array}{c} k_{0}\;\mathrm{if}\;0<R\leq R_{c}-a_{Ps}\\ B^{2}\;k_{0}\;\mathrm{if}\;R_{c}-a_{Ps}<R\leq R_{c} \end{array}\right. \end{array}$$

and, finally, by solving the integral

(31) $$k\;=\;k_{0}\;\left(4\pi /3\tilde{V}\right)\;\left[\left(1-B^{2}\right)\;{\left(R_{c}-a_{Ps}\right)}^{3}\;+\;B^{2}R_{c}^{3}\right]\;,$$

where $k_{0}=1/\pi a_{Ps}^{3}$ is the free-Ps contact density. Being the normalization coefficient B<1, this result demonstrates that the contact density is effectively lower with respect the free-Ps value. This is a fundamental consequence of the fact that, although the electron is taken strictly confined in the cavity, the positron can effectively explore the external region, especially if the electron is located near the wall. Therefore, this model is able to connect the lowering of the contact density with the geometrical properties of the wavefunction with different confinement requirements for the two particles.

The pick-off rate can be derived from a generalization of the formula of Equation (2) [10]:

(32) $$\lambda_{\mathrm{po}}\;≃\;\bar{\lambda }\;\frac{\rho_{e}}{k_{0}}\;P_{out}^{+}\;,$$

where $\rho_{e}$ is the bulk electron density, assumed uniform, and generally lower than $k_{0}$, and where $P_{out}^{+}$ is the probability of finding the positron in the volume $V_{ext}$ external to the cavity:

(33) $$P_{out}^{+}\;=\;\int_{V_{ext}}{\left|\Psi_{+}\left(r_{p}\right)\right|}^{2}\;d^{3}r_{p}\;,$$

where $\Psi_{+}\left(r_{p}\right)$ is the positron wavefunction. Within this model, the positron wavefunction square modulus can be obtained by integrating over R the relative wavefunction $|\tilde{\varphi }{\left(r,R\right)|}^{2}$, taking into account the two conditions $|r_{e}|<R_{c}$ for the confined electron and $|r_{p}|>R_{c}$ imposed by the volume $V_{ext}$.

Furthermore, for the calculation of $\lambda_{\mathrm{po}}$, it is necessary to give an evaluation of the electron density $\rho_{e}$ in the external region where the positron can be found. This quantity is usually hard to estimate because it can specifically depend on the physical properties of the material. As suggested by the analysis carried out in [19], the interaction between the positron and the electrons basically happens with electrons in the outer atomic shells, essentially *s* and *p* electrons in molecular solids, which are among the most-studied cases. For simplicity, it can be assumed that these electrons can be treated approximatively as an uniform density spread over the molecular volume. A method to obtain suitable expressions for molecular volumes is proposed in [11]: it consists in representing each atom belonging to the molecule as a sphere with the so-called *Van der Waals radius*. This permits the evaluation of the effective electron density $\rho_{e}$ for each material under study, and, finally, determines $\lambda_{\mathrm{po}}$ which, together with the contact density information, can be compared with experimental data, as effectively performed in [11].

## 5. Electron Exchange Correlation Effects

As discussed in Section 2, in the theoretical analysis of Ps confinement in porous materials, it is customary to assume that the interaction of Ps with the electrons belonging to the cavity can be treated as a small perturbation. However, this simple tenet does not always turn out to be suitable. But, on the other hand, a more refined description of these interactions can be made very complicated by the physical requirement of full electron indistinguishability. This consideration suggests that it is not fully justified to treat Ps in terms of a separate object with respect to the surrounding material, and where the Ps electron has a different status with respect to the outer electrons, especially because of the obvious direct relation with the pick-off annihilation.

In this section, we propose a method to cope with this problem by providing a theoretical framework in which we introduce perturbatively in a natural way the electron indistinguishability in the description of Ps interaction with the surroundings electrons. This can be accomplished by explicitly taking into account spin configurations and positron–electron correlations, and by performing a perturbative calculation of annihilation rates [38,39]. To understand the need of this theory, we primarily concentrate on a particular aspect of the problem called “over-counting”, which is encountered in the description of pick-off annihilation processes in cavities. After this discussion, to the aim of deriving formal expressions of the total annihilation and pick-off rates for different Ps configurations, we resort to application of the so-called *symmetry-adapted perturbation theory* (SAPT) [40], a tool especially developed to account for electron indistinguishability. Furthermore, we also consider the use of the *local density approximation* (LDA) as a method to describe the properties of the electron system. With this modelization, it is possible to explain both the pick-off processes and the lowering of the contact density. Moreover, it is possible to provide a relatively simple method for analysing the full spectrum of a PALS experiment.

### 5.1. Pick-Off and Over-Counting

Let us reconsider the general expressions describing *o*-Psand *p*-Psmeasurable annihilation rates, discussed in Section 2:

(34) $$\begin{array}{cc} \lambda_{t}\; & =\;k_{r}\;\lambda_{3\gamma }+\lambda_{\mathrm{po}}\;,\\ \lambda_{s}\; & =\;k_{r}\;\lambda_{2\gamma }+\lambda_{\mathrm{po}}\;. \end{array}$$

where $\lambda_{\mathrm{po}}=P_{\mathrm{out}}\bar{\lambda }$. The relative contact density $k_{r}$ is usually associated with possible modifications of the Ps internal structure, while the pick-off annihilation rate $\lambda_{\mathrm{po}}$ is assumed equal for both the annihilation rates $\lambda_{t}$ and $\lambda_{s}$, depending only on some geometrical probability of finding the positron outside the cavity [11,12,25].

The fact that *o*-Psand *p*-Pscondivide the same $\lambda_{\mathrm{po}}$ means that the pick-off annihilation processes are not influenced by the spin configuration of the electron of Ps in the layer surrounding the cavity, a common assumption present in the literature and in models discussed in previous sections. In this picture, exchange correlation effects (due to the Pauli exclusion principle) between the electron of Ps and the outer electrons of any spin configuration do not have any “shielding” effect on the Ps behaviour. As a consequence, the positron is free to annihilate with all the electrons present in the surrounding material, independently from their spin, and this corresponds to an over-counting of annihilation processes inside the surface region. On the contrary, if a shielding effect is considered, the external electron spin distribution will be imbalanced due to strong repulsive Pauli exchange forces with the Ps–electron, which will tend to push away electrons with the same spin. Hence, the positron will most likely annihilate only with those electrons having opposite spin with respect to the Ps–electron.

The assumption of no shielding effect never received full justification, nor was it properly discussed from a theoretical point of view. The observation that spin exchange effects can lead to different pick-off annihilation rates for *o*-Psand *p*-Pshas been remarked in [41], but never further investigated. On the other hand, for an experimental point of view it represents a minor problem since the over-counting has in fact a negligible effect on the total annihilation rate of the *o*-Pssystem, which is the longest component of PALS spectra and, therefore, is easily measurable, because usually $\lambda_{\mathrm{po}}\gg \lambda_{t}$. But this statement is not true for *p*-Ps, because pick-off and intrinsic annihilation rates may be comparable.

The question of whether the over-counting can be legitimate or not, given its strong connection to electron indistinguishability, can find an answer only in the framework of many-body quantum mechanics. A schematic treatment of the many-body Ps environment system will be given in the following subsections. As a main result, we will show that in fact pick-off is indeed different for *o*-Psand *p*-Ps. It is important to to remark that this contention does not contrast with Equation (34), since these equations can be rephrased in the following different form:

(35) $$\begin{array}{cc} \lambda_{t}\; & =\;\lambda_{3\gamma }+\left[\left(k_{r}-1\right)\;\lambda_{3\gamma }+\lambda_{\mathrm{po}}\right]\;\\ \lambda_{s}\; & =\;\lambda_{2\gamma }+\left[\left(k_{r}-1\right)\;\lambda_{2\gamma }+\lambda_{\mathrm{po}}\right]\;. \end{array}$$

Here, the term in square brackets represents the the pick-off, which is the overall contribution to the annihilation due to the external electrons. In the following, we will derive a formula that has exactly the same form.

### 5.2. Elements of the Theoretical Treatment for the Ps Environment System

A description of the quantum state of Ps (an electron–positron pair) trapped into a void must be focused on with the following considerations. In the inner part of the free-space region of the cavity, Ps can be considered an isolated bound state. Moving towards the outer part, it transforms slowly into a so-called *spur state* (sometimes called quasi-Ps) [42], essentially a positron which interacts with the full many-body electron environment. It is clear that the complexity of modelling this description arises from the fact that in the inner scenario one deals with a separate Ps–electron, while in the outer one we have to consider the complete electron indistinguishability.

The transition between these two limiting situations can be described with a suitable theoretical treatment, formulated by taking inspiration from methods used in both physics and chemistry, in particular by writing the global ground state wavefunction $\psi^{\left(0\right)}$ of the different subsystems (in our case Ps and the environment) in a factored form. Generally speaking, the interaction $\hat{V}$ between these subsystems can be considered usually small compared with the proper interaction internal to the separated noninteracting subsystems. Hence, some sort of perturbative treatment for determining the energy contribution is naturally suggested [43]. To take into account correctly the electronic antisymmetry problem, a wide range of symmetry-adapted perturbation theories (SAPT) were proposed in the literature [40]. An accurate review of SAPT theories is beyond the scope of the present discussion, and can be found, for example, in [44].

In all SAPT formulations, the first-order correction to the energy of a composite system can be determined as

(36) $$E^{\left(1\right)}=\;\frac{\langle \psi^{\left(0\right)}|\hat{V}|A\psi^{\left(0\right)}\rangle }{\langle \psi^{\left(0\right)}|A\psi^{\left(0\right)}\rangle }\;,$$

where $\hat{V}$ is a suitable interacting potential operator, while A is an antisymmetrizer operator, defined by:

(37) $$A=\frac{1}{N!}\sum_{p}{\left(-1\right)}^{p}P\;.$$

In this formula, *P* represents the permutation operator of *N* electrons, while ${\left(-1\right)}^{p}$ represents the parity of the permutation. Note that with this definition, A is idempotent, that is $A^{2}=A$. The factor $\langle \psi^{\left(0\right)}|A\psi^{\left(0\right)}\rangle =\langle \psi^{\left(0\right)}A|A\psi^{\left(0\right)}\rangle$ at the denominator of Equation (36) is the so-called intermediate-normalization condition [44].

A general formulation of a Hamiltonian for a system consisting of a Ps atom weakly interacting with an *N*–electron environment can be schematized as a sum of a free-Ps Hamiltonian ${\hat{H}}_{\mathrm{Ps}}^{\left(0\right)}$, a Hamiltonian for the surrounding material ${\hat{H}}_{b}$ and the interaction potential acting between the two subsystems:

(38) $$\hat{H}={\hat{H}}_{\mathrm{Ps}}^{\left(0\right)}\left(r_{p},r_{e}\right)+{\hat{H}}_{b}\left(r_{1},r_{2},\cdots ,r_{N}\right)+\sum_{i=1}^{N}{\hat{V}}_{\mathrm{int}}\left(r_{p},r_{e},r_{i}\right)\;,$$

where ${\hat{V}}_{\mathrm{int}}\left(r_{p},r_{e},r_{i}\right)={\hat{V}}_{C}\left(r_{e},r_{i}\right)-{\hat{V}}_{C}\left(r_{p},r_{i}\right)$. The symbols ${\hat{V}}_{C}\left(r_{x},r_{y}\right)$ indicate the general expression of the Coulomb potential between the particles symbolized by *x* and *y*, having spatial coordinates $r_{x}$ and $r_{y}$, respectively. Regarding the spin coordinates σ of the particles, for the sake of simplicity in writing, in the following we will denote with $p=(r_{p},\sigma_{p})$ and $e=(r_{e},\sigma_{e})$ the complete spin–spatial coordinates of the positron and of the Ps–electron, respectively, while with simple numbers we refer to the spin–spatial coordinates of the *N* other electrons (see, for example, the later Equation (40).

For the well-known description of Ps in porous materials, the most important feature of the interactions is the confining effect. This can be well formalized by an effective potential ${\hat{V}}_{\mathrm{eff}}\left(r_{p},r_{e}\right)$, involving only the spatial coordinates of the Ps atom components. For example, in the simple case of the TE model discussed in Section 3.1, this potential is considered an infinite quantum well ${\hat{V}}_{\mathrm{eff}}\left(r_{p},r_{e}\right)={\hat{V}}_{\infty }\left(R\right)$, hence only acting on the Ps centre of mass R. It is useful to include ${\hat{V}}_{\mathrm{eff}}$ in the definition of an effective Ps hamiltonian as ${\hat{H}}_{\mathrm{Ps}}={\hat{H}}_{\mathrm{Ps}}^{\left(0\right)}+{\hat{V}}_{\mathrm{eff}}$, so that Equation (38) can be rewritten as

(39) $$\hat{H}={\hat{H}}_{\mathrm{Ps}}\left(r_{p},r_{e}\right)+{\hat{H}}_{b}\left(r_{1},r_{2},\cdots ,r_{N}\right)+\hat{V}\;,$$

with an interaction potential $\hat{V}$ given by

$$\hat{V}=\left[\sum_{i=1}^{N}{\hat{V}}_{\mathrm{int}}\left(r_{p},r_{e},r_{i}\right)-{\hat{V}}_{\mathrm{eff}}\left(r_{p},r_{e}\right)\right].$$

By neglecting the interaction potential, the Hamiltonian of the above Equation (39) becomes separable. Its ground state can be expressed as the product of a Ps wavefunction $\Psi_{jm}$ times the antisymmetric ground state ϕ of the *N*–electron system:

(40) $$\psi_{jm}^{\left(0\right)}\left(p,e,1,\cdots ,N\right)=\Psi_{jm}\left(p,e\right)\;\phi \left(1,2,\cdots ,N\right)\;,$$

where the indexes j,m indicate the Ps spin *S* and spin projection $S_{z}$ quantum numbers (j=1 for *o*-Psand j=0 for *p*-Ps). This wavefunction is by construction antisymmetric with respect to the exchange of any two electrons 1,…,N in the material, following the relation

(41) ϕ(1⋯i⋯j⋯N)=−ϕ(1⋯j⋯i⋯N)∀i,j,

but it is not antisymmetric with respect to the exchange with Ps electron.

It is useful now to introduce some physical quantities, well known in the framework of the theory of many-body systems. The first is the external electron density, which can be derived from the square modulus of the *N*–electron normalized wavefunction and can be expressed by

(42) $$n\left(r\right)=N\sum_{\sigma_{1}}\int {\left|\phi \left(r,\sigma_{1},2,\cdots ,N\right)\right|}^{2}\;d2\ldots \;dN\;.$$

Here and in the following, we use the compact notation $\int \;di=\sum_{\sigma_{i}}\int \;d^{3}r_{i}$ to represent both spin summation and spatial integration.

Other important quantities are the so-called reduced density matrices (RDM), which are powerful concepts used in many body physics offering a convenient way for the description of the internal structure of the system of *N* indistinguishable particles. The first, and simplest, RDM is the one-body reduced density matrix (1RDM), defined as

(43) $$\Gamma^{\left(1\right)}\left(x;y\right)=N\int \phi \left(x,2,\cdots ,N\right)\phi^{*}\left(y,2,\cdots ,N\right)\;d2\cdots \;dN\;,$$

where (as defined before) *x* and *y* denote the couple of spin–spatial coordinates $\left(r_{x},\sigma_{x}\right)$ and $\left(r_{y},\sigma_{y}\right)$. Similarly, the second useful quantity is the two-body reduced density matrix (2RDM), defined as

(44) Γ(2)(x,x′;y,y′)=N2∫ϕ(x,x′,3,⋯,N)ϕ*(y,y′,3,⋯,N)d3⋯dN.

Both 1RDM and 2RDM can be written as a sum of spin components (4 in the case of 1RDM and 16 in the case of 2RDM) depending only on spatial variables, by a suitable expansion over a complete set of spin functions. Moreover, by integration over spin variables both 1RDM and 2RDM define a corresponding spatial counterpart (more details in [38]).

Note that the diagonal part of the spatial 1RDM turns out to be exactly the electron density defined in Equation (42):

(45) $$n\left(r\right)=\Gamma^{\left(1\right)}\left(r;r\right)=n_{↑}\left(r\right)+n_{↓}\left(r\right)\;,$$

where it is possible to show that this density can be explicitly separated in the two local spin up(down) density $n_{↑}$($n_{↓}$). On the other hand, the analogous diagonal part of the spatial 2RDM, $\Gamma^{\left(2\right)}\left(r_{x},r_{y};r_{x},r_{y}\right)=P\left(r_{x},r_{y}\right)$, corresponds to the pair distribution function proportional to the conditional probability of having an electron in $r_{y}$ given another one in $r_{x}$. Assuming, reasonably, that the correlation between two electrons vanishes at long distances, in this limit one has naturally that $P(r_{x},r_{y})$ satisfies the following condition:

(46) $$P\left(r_{x},r_{y}\right)\approx n\left(r_{x}\right)n\left(r_{y}\right)\;\mathrm{when}\;\left|r_{x}-r_{y}\right|\to \infty \;.$$

Regarding the opposite limit, in real systems the probability of having two electrons spatially very close to each other is strongly suppressed by the Pauli exclusion principle, and by the strong Coulomb repulsion.

Let us observe that when Ps approaches the outer part of the cavity, its wavefunction will begin to “overlap” with that of the electronic external system. Hence, exchange correlation effects can play a relevant role. Then, it is useful to introduce a suitable real parameter *S* to quantify this overlap. After calculation, it turns out that *S* is given by the following Formula [38]:

(47) $$S\;=\;\int \Psi_{jm}^{*}\left(p,e\right)\Psi_{jm}\left(p,1\right)\;\Gamma^{\left(1\right)}\left(e;1\right)\;\;dp\;de\;d1\;.$$

As a matter of fact, assuming a Ps atom confined in a void region, the interaction with the outer electrons will take place only in a very limited surface domain. Therefore, the support of the integral in this exchange overlap Equation (47) is small by construction, and in particular one has S<1.

### 5.3. Perturbative Approach to Annihilation Rates

In the literature and within a hamiltonian framework, annihilation phenomena can be treated in a simpler way by introducing an effective absorption potential $-i\hbar \hat{\lambda }/2$, where $\hat{\lambda }$ is a suitable loss rate operator [45]. It is easy to see that this potential leads to an exponential decay of the positron (or Ps) wavefunction, then accounting for particle loss with a rate that can be related with that determined via PALS experiments. This description of the annihilation process is obviously connected to the imaginary part of the energy of the system. In particular, in the perturbative description, from the imaginary part of the first-order correction to the energy one can derive a first-order correction to the annihilation rate. This correction can be calculated in the SAPT framework by using Equation (36), depending only on the knowledge of the unperturbed ground state of the system.

We start by splitting the loss rate operator into two different parts. The first part is ${\hat{\lambda }}_{e}$, which is related only to the intrinsic annihilation of the positron with the Ps–electron *e*. The second part is $\sum_{i=1}^{N}{\hat{\lambda }}_{i}$, which is related to pick-off annihilations of the positron with the system of the other *N* electrons. With this position, the Hamiltonian operator (39) is reformulated as:

(48) $$\hat{H}\;=\;{\hat{H}}_{\mathrm{Ps}}\left(r_{p},r_{e}\right)-i\frac{\hbar }{2}{\hat{\lambda }}_{e}+{\hat{H}}_{b}\left(r_{1},\cdots ,r_{N}\right)+\left[\hat{V}-i\frac{\hbar }{2}\sum_{i=1}^{N}{\hat{\lambda }}_{i}\right].$$

The expression of the pick-off annihilation operator is given explicitly by [13]:

(49) $${\hat{\lambda }}_{i}\;=\;8\pi a_{0}^{3}\;\;\delta^{3}\left(r_{p}-r_{i}\right)\;\left[\frac{1-\Sigma_{p,i}}{2}\lambda_{2\gamma }+\frac{1+\Sigma_{p,i}}{2}\lambda_{3\gamma }\right]\;,$$

where $8\pi a_{0}^{3}$ is the inverse contact density of Ps in vacuum ($1/k_{0}$), $r_{p}$ and $r_{i}$ are positron and electrons coordinates, respectively, and $\Sigma_{p,i}$ is the spin exchange operator. With this definition, it is clear that $\hat{\lambda }$ is a *contact operator*, being a linear superposition of delta functions of the positron–electron distance. The spin exchange operator Σ guarantees that the singlet Ps antisymmetric spin state annihilates via 2γ emission, while the triplet symmetric spin state annihilates via 3γ emission. This form of ${\hat{\lambda }}_{i}$ gives the correct annihilation rates for *p*-Psand *o*-Psstates in vacuum.

The total Ps annihilation rate can be straightforwardly calculated as the sum of two contributions:

(50) $$\lambda \;=\;\lambda^{\left(0\right)}+\lambda^{\left(1\right)}\;.$$

The zero-order term $\lambda^{\left(0\right)}$ is the intrinsic annihilation rate and has the explicit form

(51) $$\begin{array}{cc} \lambda^{\left(0\right)} & =\langle \psi_{jm}^{\left(0\right)}|{\hat{\lambda }}_{e}|\psi_{jm}^{\left(0\right)}\rangle \\ \; & =8\pi a_{0}^{3}\;\int dp\;{\left|\Psi_{jm}\left(p,p\right)\right|}^{2}\times \left\{\begin{array}{ccc} \; & \lambda_{2\gamma } & \mathrm{if}\;j=0\;\left(p\text{-}\mathrm{Ps}\right)\\ \; & \lambda_{3\gamma } & \mathrm{if}\;j=1\;\left(p\text{-}\mathrm{Ps}\right) \end{array}\right. \end{array}$$

which of course does not depend on the external electrons. The first-order correction $\lambda^{\left(1\right)}$ represents the (perturbative) pick-off contribution and is determined by using Equation (36):

(52) $$\lambda^{\left(1\right)}=\frac{\langle \psi_{jm}^{\left(0\right)}|\sum_{i=1}^{N}{\hat{\lambda }}_{i}|A\psi_{jm}^{\left(0\right)}\rangle }{\langle \psi_{jm}^{\left(0\right)}|A\psi_{jm}^{\left(0\right)}\rangle }\;.$$

Calculation of the normalization factor using the definition given in Equation (37), gives the following result:

(53) $$\langle \psi_{jm}^{\left(0\right)}|A\psi_{jm}^{\left(0\right)}\rangle \;=\;\frac{N}{N+1}\;,$$

where the factor N depends only on the exchange overlap *S* as N=1−S. The factor (N+1) at the denominator represents the total number of permutations of the Ps electron with the *N* electron system.

Regarding the physical meaning of these formulas, it is important to observe that the antisimmetrized wavefunction $|A\psi_{jm}^{\left(0\right)}\rangle$ describes in a natural way positron–electron correlation factors for all surrounding electrons by definition through suitable expressions of the two-particle Ps wavefunction $\Psi_{jm}\left(p,e\right)$. On the contrary, the correlation between the Ps–electron with the external electrons is not explicitly considered in this formulation, since its contribution to annihilation is negligible.

For the sake of definiteness, from now on we will concentrate on a particular component of *o*-Ps, by fixing {jm}={11}. Anyway, a similar calculation can be performed for any Ps angular quantum numbers. By rewriting Equation (52) by putting N on the left side and using the antisymmetric property of ϕ, it possible to show in general that the overall correction to the annihilation rate can be written as the sum of three different contributions:

(54) $$N\lambda^{\left(1\right)}\;=\;\lambda_{\mathrm{sym}}\;+\lambda_{\mathrm{ex}}\;+\lambda_{\mathrm{corr}}\;,$$

whose explicit expression can be found in [38]. The cumbersome calculation involves the electron density and the 1RDM and 2RDM representations over spin components, as defined in Equations (42)–(45). with the assumption of uniform spin distribution for the system of external electrons, implying $n_{↑}\left(r\right)=n_{↓}\left(r\right)=\frac{1}{2}n\left(r\right)$.

The first term $\lambda_{\mathrm{sym}}$ represents the direct contribution to the annihilation with external electrons, and retains exactly the same expression for *o*-Psand *p*-Ps, i.e., it is fully symmetric with respect to Ps spin configuration, similarly to what was discussed previously about the pick-off annihilation rate (see Equation (34)). As a matter of fact, $\lambda_{\mathrm{sym}}$ turns out to be proportional to the averaged annihilation rate $\bar{\lambda }$ defined in Equation (3). The second and last terms $\lambda_{\mathrm{ex}}$ and $\lambda_{\mathrm{corr}}$ in Equation (54) are exchange contributions to annihilation. In the calculation of $\lambda_{\mathrm{ex}}$, the annihilation operator was directly acting on the Ps spin wavefunction, so that one obtains in fact diverse final expressions for the different spin configurations *o*-Psand *p*-Ps. This exchange contribution $\lambda_{\mathrm{ex}}$ can be expressed in a simple form using the definition of the one-body reduced density matrix (1RDM) of Equation (43). In the case of *o*-Ps, it is proportional to $\lambda_{3\gamma }$, while for *p*-Psit is proportional to $\lambda_{2\gamma }$. Finally, the last term in Equation (54) is an exchange–correlation contribution to annihilation, and it is related to the two-body reduced density matrix $\Gamma^{\left(2\right)}$ of the system and to the Ps spin configuration.

Now, having established the formal basic expression for the annihilation rates, we discuss this achievement to provide more physical insight. Regarding the system interacting with Ps, we made the only reasonable assumption of uniform spin distribution, a condition affirming the absence of a local spin polarization in the unperturbed ground state of the electronic system near the cavity region. On the other side, no assumption on the form of ϕ has been carried out, so that the result obtained in Equation (54) about the annihilation rate is completely general. But to obtain more manageable results it is necessary to introduce further approximations.

In particular, we consider the so-called local density approximation (LDA). Under the LDA framework, the properties of an electronic system with a density profile n(r) are locally modelled in the same manner of those of a free electron gas with the same density. Within this framework, it can be shown that the 1RDM has an analytical expression [46], essentially proportional to the density profile:

(55) $$\Gamma^{\left(1\right)}\left(x;y\right)=\delta_{\sigma_{x}\sigma_{y}}\frac{n(R_{xy})}{2}\;B\left(k_{F}\left(R_{xy}\right)\;\left|r_{xy}\right|\right)\;,$$

where $k_{F}\left(R_{xy}\right)={\left(3\pi^{2}n\left(R_{xy}\right)\right)}^{1/3}$ is a “local” Fermi momentum and

(56) $$B\left(x\right)=3\frac{\sin(x)-x\cos(x)}{x^{3}}\;.$$

In these equations, we used the notation $R_{xy}=\left(r_{x}+r_{y}\right)/2$ and $r_{xy}=r_{x}-r_{y}$, which are the average and the relative position of the two particles *x* and *y*, respectively. But in the general case, a similar simple result does not hold for 2RDM because this quantity strongly depends on the system under examination. This fact is particularly relevant for the calculation of $\lambda_{\mathrm{corr}}$. However, it is possible to demonstrate that for ordinary values of n(r) this quantity is in fact a higher order contribution to the annihilation rates [38]. Therefore, given that we are considering only first-order corrections to annihilation rates, we can neglect $\lambda_{\mathrm{corr}}$ from now on.

The exchange overlap *S* and all the corrections to the annihilation rates can in principle be determined by using the definitions just introduced, in the case in which the electron density function n(r) and the Ps spatial wavefunction can be expressed in an explicit form. We note that the theory presented here can be developed starting from any given Ps and electron bulk wavefunctions, in particular accommodating any suitable choice of the spatial part of the Ps wavefunction and any form of positron–electron correlation factors, as usually carried out in the literature [31,47]

### 5.4. Calculation of Pick-Off Annihilation Rate

In order to find a suitable expression of the spatial Ps wavefunction $\Psi (r_{p},r_{e})$, it is necessary to particularize the form of the hamiltonian, in particular by choosing some suitable effective potential ${\hat{V}}_{\mathrm{eff}}$ acting on the two particles, as discussed in previous subsections. To quote an example and for the sake of simplicity, with the objective of a direct comparison with the well-studied TE model, we select a spherical cavity geometry of radius $R_{c}$, and suppose that Ψ can be written in a simple factored form:

(57) $$\Psi \left(r_{p},r_{e}\right)\;=\;\psi \left(r_{pe}\right)\Psi_{\mathrm{TE}}\left(R_{pe}\right)\;,$$

depending on the relative $r_{pe}$ and centre of mass $R_{pe}$ coordinates, with usual definitions. As discussed in Section 3, the confining effect acting on Ps centre of mass is taken into account by using an infinite potential barrier located at a distance $R_{c}+\Delta$ from the cavity centre [20]. Therefore, the centre of mass ground–state wavefunction is the same as in Equation (4):

(58) $$\Psi_{\mathrm{TE}}\left(R_{pe}\right)\;=\;\frac{1}{\sqrt{2\pi (R_{c}+\Delta )}}\frac{\sin\left(\pi R_{pe}/\left(R_{c}+\Delta \right)\right)}{R_{pe}}\;.$$

Moreover, since we are neglecting all Coulomb potentials except those intrinsic of the bound Ps atom, the radial part of the relative wavefunction is assumed to be the same as to the unperturbed Ps, which is a hydrogen-like 1S orbital:

(59) $$\psi \left(r_{pe}\right)=\sqrt{k_{0}}\;\;e^{-r_{pe}/a_{Ps}}\;,$$

(as already considered in Section 4.4).

On the other hand, giving a precise expression for the electron density function n(r) can be very complicated by the fact that it is necessary to take into account all the relevant interactions which are naturally present in the system. While it is expected that electron–electron repulsion adds a negligible contribution to annihilation, the opposite is in fact true for positron–electron attraction, hence leading to an enhancement of the electron density at the positron position, which can induce an increase in the annihilation rate [48]. For simplicity, we can just define an effective electron density $\rho_{e}$ interacting with Ps in the interaction region limited by the shell layer [39], and write

(60) $$\begin{array}{c} n\left(r\right)=\left\{\begin{array}{cc} \rho_{e} & \;\mathrm{if}\;r\geq R_{c}\\ 0 & \;\mathrm{if}\;r<R_{c} \end{array}\right. \end{array}$$

which is also the exact prescription of the TE model.

Using Equations (57) and (60), and the LDA expressions in Equations (55) and (56), the symmetric contribution $\lambda_{\mathrm{sym}}$ to the annihilation and the exchange overlap *S* are easily calculated (see [38]). The important exchange correction terms $\lambda_{\mathrm{ex}}$ appropriate for the different Ps spin configurations are obtained as follows. The exchange correction $\lambda_{\mathrm{ex}}^{3\gamma }$ for *o*-Psturns out to be given by the expression

(61) $$\lambda_{\mathrm{ex}}^{3\gamma }\;=\;-\frac{\lambda_{3\gamma }\;\;\rho_{e}}{2\sqrt{k_{0}}}\int_{R_{pe}>R_{c}}\;d^{3}r_{p}\;d^{3}r_{e}\;\Psi_{\mathrm{TE}}^{*}\left(R_{pe}\right)\Psi_{\mathrm{TE}}\left(r_{p}\right)\psi \left(r_{pe}\right)B\left(k_{F}\;r_{pe}\right)\;,$$

proportional to the intrinsic annihilation rate $\lambda_{3\gamma }$ as expected. For *p*-Psthe exchange correction, $\lambda_{\mathrm{ex}}^{2\gamma }$ is simply obtained by interchanging $\lambda_{3\gamma }↔\lambda_{2\gamma }$ in the previous expression. Finally, by collecting all these first-order corrections under the scheme of Equation (54), using N=1−S and adding the unperturbed intrinsic annihilation, we obtain the formal expressions for the total annihilation rates of *o*-Psand *p*-Psin the elegant form [38,39]:

(62) $$\begin{array}{cc} \lambda_{t} & =\left[\lambda_{3\gamma }-\frac{\lambda_{\mathrm{ex}}^{3\gamma }}{1-S}\right]+\frac{\lambda_{\mathrm{sym}}}{1-S}\;,\\ \lambda_{s} & =\left[\lambda_{2\gamma }-\frac{\lambda_{\mathrm{ex}}^{2\gamma }}{1-S}\right]+\frac{\lambda_{\mathrm{sym}}}{1-S}\;. \end{array}$$

These expressions clearly demonstrate that a difference in pick-off annihilation rates between Ps spin configurations can be solely ascribed to exchange contributions.

This compact result was obtained by using some simplifying approximations, but to derive reliable results susceptible to physical interpretation, it must be noted that the spatial integrals appearing in the definitions of the various terms in the formula have no analytical expression, and one needs to use numerical methods. However, by using simple geometrical considerations some insights about the qualitative behaviour of the annihilation rates can be derived. First, we note that the greatest contribution to spatial integrals between electrons variables comes when their positions are sufficiently near to the positron position, in a sphere roughly the size of Ps. Taking again as a reference the TE picture, this size is generally small compared to the range $R_{c}+\Delta$, which coincides with the range of the possible positions of the centre of mass wavefunction $\Psi_{\mathrm{TE}}\left(R\right)$. Hence, we can approximate all the occurrences of the centre of mass positions in the integrals with a single dependence on *R*. Now, by observing that n(R) has a step behaviour (Equation (60)), it is useful to define the quantity $P_{\mathrm{out}}^{\prime }$ as the probability of finding the Ps centre of mass in the interaction region outside $R_{c}$. In spherical coordinates

(63) $$\begin{array}{cc} P_{\mathrm{out}}^{\prime } & \equiv 4\pi \int_{R_{c}}^{R_{c}+\Delta }{\left|\Psi_{\mathrm{TE}}\left(R\right)\right|}^{2}R^{2}\;dR\\ \; & =\;1-\frac{R_{c}}{R_{c}+\Delta }+\frac{1}{2\pi }\sin\frac{2\pi R_{c}}{R_{c}+\Delta }\;, \end{array}$$

which is again the well-known expression derived in the framework of the TE model because $P_{\mathrm{out}}^{\prime }$ is similar to the $P_{out}$ used there. By using the above discussed approximation in all the integrals defining the annihilation contributions, we obtain the annihilation rates for *o*-Psand *p*-Psin the explicit form similar to that presented in Section 5.1, exhibiting two separate contributions due to the pick-off processes:

(64) $$\begin{array}{cc} \lambda_{t} & =\left[1-\frac{P_{\mathrm{out}}^{\prime }A\left[\nu \right]}{1-P_{\mathrm{out}}^{\prime }C\left[\nu \right]}\right]\lambda_{3\gamma }+\left[\frac{\rho_{e}}{k_{0}}\frac{P_{\mathrm{out}}^{\prime }}{1-P_{\mathrm{out}}^{\prime }C\left[\nu \right]}\right]\bar{\lambda }\;,\\ \lambda_{s} & =\left[1-\frac{P_{\mathrm{out}}^{\prime }A\left[\nu \right]}{1-P_{\mathrm{out}}^{\prime }C\left[\nu \right]}\right]\lambda_{2\gamma }+\left[\frac{\rho_{e}}{k_{0}}\frac{P_{\mathrm{out}}^{\prime }}{1-P_{\mathrm{out}}^{\prime }C\left[\nu \right]}\right]\bar{\lambda }\;, \end{array}$$

where we have inserted two auxiliary functions *A* and *C* of the variable $\nu =2k_{F}a_{0}$, which depend on $\rho_{e}$ through $k_{F}$ [38,39]. Their values range from 0 to 1 as a function of the electron density $\rho_{e}$.

The main advantage of this final formulation is that in this way geometrical effects appear well separated from electron exchange contributions. As a matter of fact, only in the definition of the probability $P_{\mathrm{out}}^{\prime }$, which depends on the cavity radius $R_{c}$ and on the thickness of the interacting region Δ, are contained the geometrical parameters On the other hand, the effects due to electron exchange define the appropriate analytical form by which the parameters $k_{0}$, $\rho_{e}$ and the same $P_{\mathrm{out}}^{\prime }$ appear.

Within this formulation, by comparing with Equation (34) it turns out that the intrinsic relative contact density assumes the explicit expression

(65) $$k_{r}\;=\;\left[1-\frac{P_{\mathrm{out}}^{\prime }A\left[\nu \right]}{1-P_{\mathrm{out}}^{\prime }C\left[\nu \right]}\right]\;,$$

which justifies the well-known lowering of the contact density observed for Ps in nanocavities. The meaning of $k_{r}$ is that of a useful indicator of the degree of dissociation for a Ps atom interacting with the medium. In other words, it signals the separability of the Ps–electron. The overlap of Ps with surrounding electrons lowers $k_{r}$ with respect to its maximum value ($k_{r}=1$ for Ps in vacuum), and in particular $k_{r}$ tends to vanish as the original Ps state loses its identity. In the limit case of $k_{r}=0$, no distinction between *o*-Psand *p*-Pspick-off annihilation rates is possible, because all electrons (the Ps–electron and the external ones) have to be taken on equal footings. It is of paramount importance to note that, in this picture, the lowering of the contact density is essentially due to the electron indistinguishability. By no means it can be related to a spatial deformation of the Ps wavefunction, contrary to what is proposed and discussed in other models presented in previous sections.

As a final observation, it is interesting to compare directly Equation (64) with the annihilation formulas presented in Section 5.1 and with the TE results. The equivalence between the symmetric contributes to pick-off annihilation rates as predicted by the simple formula of Equation (34) and by the result in Equation (64) is simply obtained by setting:

(66) $$P_{\mathrm{out}}=\frac{\rho_{e}}{k_{0}}\frac{P_{\mathrm{out}}^{\prime }}{1-P_{\mathrm{out}}^{\prime }C\left[\nu \right]}\;.$$

Applying this condition, we can rewrite the fundamental Equation (64) as

(67) $$\begin{array}{cc} \lambda_{t} & =\left[1-\frac{k_{0}}{\rho_{e}}A\left[\nu \right]P_{\mathrm{out}}\right]\lambda_{3\gamma }+P_{\mathrm{out}}\bar{\lambda }\;,\\ \lambda_{s} & =\left[1-\frac{k_{0}}{\rho_{e}}A\left[\nu \right]P_{\mathrm{out}}\right]\lambda_{2\gamma }+P_{\mathrm{out}}\bar{\lambda }\;, \end{array}$$

which shows a clear similarity to Equation (34) and of course can be interpreted directly as in Equation (35). Since this formulation is an equivalent version of the TE model, when Ps is in the outer shell of thickness Δ≃ 0.166 nm, the intrinsic relative contact density in the surface region can be simply derived by assuming $P_{\mathrm{out}}=1$ obtaining

(68) $$k_{\mathrm{out}}=1-\frac{k_{0}}{\rho_{e}}A\left[\nu \right]\;.$$

Under the limiting situation in which the probability of having an external electron at the positron position assumes the same value as in free-Ps (i.e., when $\rho_{e}\to k_{0}$), $k_{\mathrm{out}}$ can be explicitly calculated as [38]:

(69) $$\lim_{\rho_{e}\to k_{0}}k_{\mathrm{out}}=1-0.472=0.527\;.$$

This result is very close to the expectation value of the relative contact density of *one* electron in the negative ion ${\mathrm{Ps}}^{-}\;$ [49]. As a matter of fact, this result can be expected and is not surprising because it is well known that in the usual picture of ${\mathrm{Ps}}^{-}$ only one electron is closely bound to the positron [50]. Moreover, a one–half factor in the contact density comes from the normalization of the total antisymmetric wavefunction. This interesting result suggests an intuitive simplified picture in which in the internal cavity region Ps behaves similarly to being in vacuum, whereas inside the interaction region in the external shell of thickness Δ Ps resembles a ${\mathrm{Ps}}^{-}\;$.

As another observation, it is possible to note that in the case of low electron density values, the range of the shielding effect, i.e., the tendency to Ps electron to repel external electrons with the same spin, will be enhanced and, in particular for $\rho_{e}=\rho_{0}\approx 0.3k_{0}$, the intrinsic contact density is found to vanish in the surface region:

(70) $$k_{\mathrm{out}}={\left.1-\frac{k_{0}}{\rho_{e}}A\left[\nu \right]\right|}_{\rho_{e}=\rho_{0}}=0\;,$$

so that Equation (67) become

(71) $$\begin{array}{cc} {\left.\lambda_{t}\right|}_{\rho_{0}} & =\left[1-P_{\mathrm{out}}\right]\lambda_{3\gamma }+P_{\mathrm{out}}\bar{\lambda }\;,\\ {\left.\lambda_{s}\right|}_{\rho_{0}} & =\left[1-P_{\mathrm{out}}\right]\lambda_{2\gamma }+P_{\mathrm{out}}\bar{\lambda }\;, \end{array}$$

which are of the same form of the general formulas discussed in Section 2. This final expression can give a justification to the assumptions that are employed in the family of models discussed in that section.

## 6. Conclusions

The main objective of this review is to analyse the various theories proposed for describing the features of Ps in the cavities present in solid media. This is motivated by the increase in the interest to use Ps as a valuable probe to characterize the structure of many kinds of materials, using PALS as the main technique. In order to gain information on the structural properties of the medium from PALS data, we need a satisfactory theory of Ps formation and annihilation in condensed matter. Any model giving a quantitative description of Ps trapped in the cavities of a material should be able to correctly explain the pick-off process, that is, the annihilation with an external electron belonging to the surrounding medium rather than the Ps electron.

Most of the models presently used to describe Ps trapped in sub-nanometric holes follow the TE assumption, which considers Ps as a featureless single quantum particle inside an infinite potential well. A quantitative relationship between the cavity size and the pick-off decay rate $\lambda_{\mathrm{po}}$ is obtained. Temperature effects as well as different geometries for the void have extended the initial formulation.

After having reviewed the various available models dealing with Ps caged inside (sub)nanometric voids, we pointed out that some of the annihilation features are still missing a suitable theoretical framework. The most important one, usually observed in many structures allowing Ps formation, is the intrinsic relative contact density, which is generally lowered with respect to the value in vacuum. This quantity, which represents the probability to find the electron of Ps at the positron position, is a fundamental measurable quantity of interest. In order to describe the intrinsic contact density, it is necessary to know the Ps internal structure. For this purpose various two-particle models were proposed, which consider separate degrees of freedom for the two particles forming Ps. These models introduce some effective potentials to describe the interaction of the Ps atom with the medium.

In particular, the fundamental aspect of all models is picturing Ps as affected by the confining potential of the cavity. The confining effects can be attributed, for example, to an infinite wall potential, to harmonic potentials and, especially in the case of liquids, to the formation of a bubble around the Ps with a dielectric screening. A proposal which combines the general approach of the TE model with specific chemical features of the medium is to consider directly the confining effect due to Pauli exchange forces, which can be ascribed to the Ps–electron only. On the other hand, the positron can be attracted toward the bulk due to the positive value of the positron work function as exhibited by many materials. This effect tends to pull the positron apart from the electron of Ps, so giving a partial justification for the lowering of the contact density. The solution of this model effectively demonstrates the lowering of various confining solid media supporting the experimental results.

Nevertheless, the presence of several unknown parameters and, in some cases, the extreme conditions which are necessary to account for the lowering of the contact density were unsatisfactory. As a consequence, the problem of Ps trapping in materials showing small voids has been tackled by means of a different approach, by considering the exchange interaction with the external electrons surrounding Ps. In the literature, the usual point of view is to assume small interactions. In other words, Ps is considered as weakly interacting with the local environment. It follows that a possible way forward for this study is to use a perturbative approach, even if the request of full electron indistinguishability adds a complication. Therefore, an appropriate theoretical framework can be set up by using the symmetry adapted perturbation theory (SAPT), in which Ps is considered a separate “entity” and the Ps electron has a kind of special role in comparison with the external electrons.

Using this theory, we showed that some concepts, which in the literature have been the objects of different interpretations, can be unequivocally clarified. In particular, we put into a new light the meaning of the relative contact density $k_{r}$, always correlated only to the spatial part of the wavefunction for trapped Ps, and critically discussed the form of the pick-off annihilation rate, usually considered identical for both *o*-Psand *p*-Ps. In practice, we showed that the Ps electron exerts a spin-shielding action which implies different pick-off decay rates for the two Ps sublevels. This feature has been generally misunderstood and, to the best of our knowledge, never considered in the previous works. We showed also that it is possible to recover a symmetric quasi-analytic form for the two pick-off decay rates by explicitly taking the observed lowering of the relative contact density into account.

The final model contains only three parameters: the radius $R_{c}$ of the (spherical) void, the thickness of the interaction layer Δ, and the magnitude of the external electron density as probed by the positron $\rho_{e}$. They are summarized in Equation (62), which gives the total annihilation rates for *p*-Psand *o*-Ps. The model supplies a simple justification of the lowering of the relative contact density without introducing any potential able to pull apart the two particles forming Ps, as evidenced by using the same unperturbed expression for the Ps relative wavefunction as in vacuum. The fact that $k_{r}\to 0$ for sufficiently small values of $R_{c}$ or for high values of the external electron density simply derives from a higher overlap with the surrounding electrons and does not depend on the spatial deformation of the Ps wavefunction, as generally admitted in previous models. Put in other terms, the concept of Ps itself becomes meaningless when the electrons cannot be any longer distinguished.

As a final comment, we point out that, although we used a simple expression for the Ps wavefunction, it is straightforward to extend our results to any one-particle and two-particles models which describe Ps trapped in cavities, as well as to effective theories modelling electron–positron spatial correlations, as sometimes found in the literature [31,47].

This review suggests that the proposed final model needs to be tested and validated by further studies. In this connection, PALS and magnetic quenching experiments, also in the presence of external pressures [51], should be most useful since they entail a reduction in the unknown free parameters.
